# Extracellular Particles as Carriers of Cholesterol Not Associated with Lipoproteins

**DOI:** 10.3390/membranes12060618

**Published:** 2022-06-14

**Authors:** Sergey Landa, Nicolay Verlov, Natalia Fedorova, Mikhail Filatov, Rimma Pantina, Vladimir Burdakov, Elena Varfolomeeva, Vladimir Emanuel

**Affiliations:** 1 Petersburg Nuclear Physics Institute Named by B.P. Konstantinov of NRC “Kurchatov Institute”, 1, Orlova Roshcha, 188300 Gatchina, Russia; verlov_na@pnpi.nrcki.ru (N.V.); fedorova_nd@pnpi.nrcki.ru (N.F.); filatov_mv@pnpi.nrcki.ru (M.F.); pantina_ra@pnpi.nrcki.ru (R.P.); burdakov_vs@pnpi.nrcki.ru (V.B.); varfolomeeva_ey@pnpi.nrcki.ru (E.V.); 2 Department of Clinical Laboratory Diagnostics, St. Petersburg State Medical University, I.P. Pavlova of the Ministry of Health of the Russian Federation, 9, Lev Tolstoy St., 197022 Saint Petersburg, Russia; vladimirem1@gmail.com; 3 Saint Petersburg Clinical Research and Practical Center of Specialized Types of Medical Care (Oncological), 196605 Saint Petersburg, Russia

**Keywords:** exomeres, exosomes, cholesterol, lipoproteins, dynamic light scattering

## Abstract

Exosomes and exomeres are the smallest microparticles ranging from 20 to 130 nm in diameter. They are found in almost all biological fluids. Exosomes and exomeres are of considerable interest since they can be involved in intercellular signaling and are biological markers of the state of cells, which can be used for diagnostics. The nomenclature of exosomes remains poorly developed. Most researchers try to classify them based on the mode of formation, physicochemical characteristics, and the presence of tetrasporin markers CD9, CD63, and CD81. The data presented in this work show that although exomeres carry tetrasporin biomarkers, they differ from exosomes strongly in lipid composition, especially in cholesterol content. The production of exomeres by cells is associated with the synthesis of cholesterol in cells and is expressed or suppressed by regulators of the synthesis of mevalonate, an intermediate product of cholesterol metabolism. In addition, the work shows that the concentration of extracellular particles in the body correlates with the concentration of cholesterol in the plasma, but weakly correlates with the concentration of cholesterol in lipoproteins. This suggests that not all plasma cholesterol is associated with lipoproteins, as previously thought.

## 1. Introduction

Extracellular particles (EPs) of blood plasma were discovered in 1967 by the English researcher Peter Wolf and were initially called “platelet dust” [1]. Later it was shown that when reticulocytes are cultured, vesicles called exosomes are released into the medium, the membrane of which is close to the membrane of producing cells in its composition and in the receptors presented on it. The authors suggested that the main role of these particles is to remove excess membrane proteins during the maturation of reticulocytes [2].

Currently, EPs are studied in much more detail. These particles are characterized by extreme heterogeneity and diverse origin. EPs are found in almost all biological fluids, as well as in environments in which cell cultures are cultivated. Mammalian cells are able to secrete two types of extracellular vesicles (EVs): exosomes (cleaved from the membranes of intracellular compartments, 20–100 nm in diameter) and ectosomes (also called microparticles or microvesicles, cleaved from the plasma membrane, 100–1000 nm in diameter) [3].

The nomenclature of exosomes is still poorly developed. Most researchers try to classify them by the method of formation, physicochemical characteristics (size, density, etc.), and markers [4]. For the present, vesicles formed inside multivesicular endosomal bodies with a diameter of 30–120 nm, carrying CD9, CD63, CD81 tetraspanins [5] and containing an internal cavity detected by electron [6] or atomic force microscopy [7,8] are referred to as exosomes.

However, back in 2010, we showed using the method of dynamic light scattering that the histogram of the size distribution of exosomes (PSD) is bimodal: microvesicles are divided into two fractions with average sizes of about 25 and 90 nm. Each fraction is homogeneous, which is confirmed by atomic force microscopy. The immunoaffinity method demonstrated that all microvesicles carry antigenic determinants recognized by antibodies to the main histocompatibility complex of the first type (HLA-ABC) and exosomal markers CD9 and CD63 [9].

In 2018, two subtypes of exosomes, and also particles with a size of less than 50 nm, which were given the name “exomeres”, were revealed by the method of fractionation in a force field (asymmetric flow field-flow fractionation—a4f) [10]. In addition, it was shown that exosomes exhibit a cup-shaped morphology, with a size range of 50–150 nm, while exomeres have a size of less than 50 nm and a “point” morphology [11,12]. Our studies performed by atomic force microscopy showed, firstly, the presence of a central cavity in exosomes and its absence in exomeres, and secondly, significant differences in the biomechanical properties of the central part of exosomes and exomeres [13].

The membranes of exosomes and especially exomeres are enriched with cholesterol, sphingomyelin, and ganglioside GM3 [14]. However, exomeres are very different from exosomes in terms of protein and lipid composition. The exomeres are enriched with proteins involved in metabolism (for example, hexokinase 1 (HK1), glucose-6-phosphatizomerase (GPI) aldolase A (ALDOA), glutamine-oxalic acetic transaminase 1 and 2 (GOT1), (GOT2), and fumate hydratase (FH)), as well as with some heat shock proteins from the HSP90 family. In addition, it was shown that phospholipids and sphingolipids predominate among the lipids of exomeres. Exomeres in relation to exosomes are much more enriched with esterified cholesterol: the ratio of esterified cholesterol in exomeres to exosomes is approximately 4 to 1, while the ratio of unesterified cholesterol in exosomes to exomeres is 10:1 [14]. This is also indicated by our data on the difference in the biomechanical properties of exosome and exomere membranes [13].

Along with studies of exosomes and exomeres, there are a fairly large number of papers devoted to extracellular vesicles of apoptotic origin (ApoEV). They are a group of subcellular extracellular vesicles formed during the decay of dying cells [15,16]. Apoptotic vesicles can be classified by diameter: larger membrane-wrapped vesicles called ApoBD/AB have diameters of 1000–5000 nm [17] and smaller vesicles called apoptotic microvesicles (ApoMVs) or exosome-like ApoEV [18] have diameters of 50–1000 nm [19,20].

The above data made it necessary to test the assumption of whether the production of exosomes and (or) exomeres is related to cell death.

The purpose of this work was to study exomeres and exosomes, to identify their common properties and the features of exomeres, as well as to make an attempt to identify at least some metabolic processes in the cell and the whole organism that lead to the formation of exomeres.

## 2. Materials and Methods

*Cell cultures and cultivation conditions.* Transplanted human cell cultures were used: Gl-Tr was obtained by us from glioblastoma, A172—human glioblastoma was obtained from the Collection of Cell Cultures of the Institute of Cytology, Russian Academy of Sciences, St. Petersburg, Russia.

The cells were cultured in DMEM/F12 medium (Biolot) supplemented with 10% fetal serum (ESC) (Biolot) without antibiotics, in an atmosphere of 5% CO_2_, at 37 °C until a monolayer was formed. Then, the culture medium produced by the cells was replaced with a fresh one, while the percentage of ESCs was reduced to 5% and cultivated until the amount of EP ceased to increase (usually 3–4 days) (see Supplement Figure S1). If the use of fetal serum was unacceptable, the cells were washed three times with phosphate buffer (PBS) and embedded in DMEM/F12 basic medium supplemented with Cholesterol Lipid Concentrate 250× (Gibco) at the rate of 1 mL of concentrate per 250 mL of medium and cultured as described above.

Samples of the conditioned nutrient medium (CM) were taken once a day for analysis by the DLS method. After that, the BM samples were centrifuged at 2000× *g* for 20 min and 10,000× *g* for 30 min, filtered through a disposable Minisart High Flow filter with a polyethersulfone membrane with a pore diameter of 0.1 μm (Sartorius, Göttingen, Germany, cat. no. 16553K) to remove dead cells and their fragments, and DLS studies were undertaken to detect the presence of EPs.

*Biological fluids*. In the cases of in vivo studies, the source of EPs was blood serum obtained from 41 donors. Donors were selected from patients of the University Clinic of Pavlov First Saint Petersburg State Medical University who received a referral from their attending physician for a lipid profile analysis. All donors gave informed consent to participate in scientific research. They were informed about the purpose and significance of the results that will be obtained during the research. The research was approved by the Ethics Committee of NRC “Kurchatov Institute”—PNPI (Protocol № 05-2020/LEC from 30.04.2020). The group of donors was specially formed to be heterogeneous in order to obtain the maximum spread of the studied parameters. The only condition for not being included in the group was the taking of statins by the donor at the time of the start of the studies. When the attending physician prescribed statin therapy to the donor (rosuvastatin 10 mg/day) after 21 days, the donor donated blood for the second analysis. There were 21 such cases. Blood was taken from a vein and placed into vacuum tubes with a coagulation activator according to a standard algorithm [21]. Determination of the content of lipoproteins in plasma was carried out according to the standard method (0.5 mL was taken from the supernatant for the DLS analysis). Before the analysis, plasma samples were centrifuged in the 10,000× *g* mode for 30 min and filtered through a disposable Minisart High Flow filter with a polyethersulfone membrane with a pore diameter of 0.1 microns (Sartorius, Germany, Cat No. 16553K) to remove dead cells and their fragments.

*Reagents, antibodies, and dosage forms.* Reagents from the following manufacturers were used in the work: Triton X-100-PanReac—AppliChtm (Barcelona, Spain), Saponin—SERVA (Heidelberg, Germany), methylbetacyclodextrin—Chemical Line (Saint-Petersburg, Russia), low-, very-low-, and high-density lipoproteins—Sigma-Aldrich, polyclonal antibodies—Cloud-Cline Corp. (Katy, TX, USA), Levemir^®^ Novotec insulin Bagsward, Denmark.

*The method of dynamic light scattering.* The measurements were carried out using a laser correlation spectrometer DLS (INTOX MED LLC, St. Petersburg, Russia) with a heterogeneous measurement scheme [22]. Mathematical processing of the obtained data was carried out using the algorithm [22] using the QELSspec (version 3.4) software package, Gatchina, Russia.

*Identification of surface biomarkers of extracellular particles.* To determine the presence of specific surface markers on particles of different sizes, a combination of immunosorption and DLS was used, as described and checked by an independent method earlier [23].

### Apoptosis

Cells (GL-Tr, A172, FLEH) were cultured in 6-well plates at 37 °C in a 5% CO_2_ incubator in DMEM/F12 medium (Biolot, Saint-Petersburg, Russia) supplemented with 10% fetal bovine serum (FBS) until the formation of a monolayer. Cell death can be induced by adding hydrogen peroxide to the culture medium [24]. Then, H_2_O_2_ was added at concentrations of 1.8 mM for 4 h. Cells without added H_2_O_2_ were used as an experimental control. After that, cells were disaggregated with trypsin: Versene Solution (1:1) (Biolot, Saint-Petersburg, Russia) and centrifuged. The “Annexin-FITC” kit (Beckman Coulter, Indianapolis, IN, USA) was used according to the manufacturer’s protocol. At least 20,000 cells were analyzed in each experiment. Annexin V-FITC and PI double staining were regarded as apoptotic or necrotic cells. The cytometer Cell Lab Quanta Beckman Coulter (Indianapolis, IN, USA) was used for fluorescence registering.

*Treatment of samples with detergents. Obtaining the curves of EPs’ lysis.* Since the effective concentration of Triton X-100 causing 100% lysis of HeLa cells is 0.2 mM [25], and EPs are more resistant to the action of Triton X-100, this detergent was added in the amount necessary to obtain the final concentration of 2 mM/L. The final concentration of saponin was 0.08%. Detergents were added directly to the cuvette with the sample. The measurements allowing one to fix the distribution (PSD) before the action of the detergent begins were carried out immediately after the addition of the detergent at a temperature of 4 °C, in order to block its action if possible. The repeated measurement was carried out after incubation of the samples for 1 h at 37 °C. After that, the obtained histograms (PSD) were compared. To plot the lysis curve, Triton X-100 was added to the samples in the amount necessary to obtain the final concentration of 0.2, 1, 2, 5, 10 mM/L and incubated for 1 h at 37 °C, and then measurements were carried out by DLS method. Similarly, the lysis curves were obtained from measurements of samples with Triton X-100 paired with methyl beta-cyclodextrin (MβCD). The final concentration of MβCD was 5 mM/L for all samples.

*Study of the dynamics of EPs production and the effect of insulin on it.* We used the dosage form of insulin detemir, the drug named Levemir. Levemir^®^ is produced by recombinant DNA biotechnology using the *Saccharomyces cerevisiae* strain. Detemir is a soluble basal analogue of human-prolonged-acting insulin with a flat action profile. The action profile of insulin detemir is significantly less variable compared to insulin isophane and insulin glargine. The duration of action of the drug is up to 24 h, which provides the possibility of a single daily administration [26]. We chose the insulin dose based on the fact that the concentration of insulin in the blood plasma is on average 6–27 mIU/mL (0.218–1 mcg/mL) [27]. Therefore, we injected insulin into the culture medium so that its final concentration was 1 mcg/mL and corresponded to its maximum concentration in blood plasma (27 mIU/mL).

The cells were cultured in DMEM/F12 medium (Biolot), with the addition of 10% embryonic serum (FBS) (Biolot), without antibiotics, in a 5% CO_2_ atmosphere, at 37 °C for 7 days. The cells were cultured in three ways: without the addition of insulin, with the addition of insulin before cultivation, and with the daily addition of insulin. CM samples for analysis by the DLS method were taken one time per day. In the third variant of cultivation, immediately after sampling for analysis, insulin was added to the CM at the rate that its final concentration was 1 mcg/mL. The selected CM samples were centrifuged in the mode of 2000× *g* for 20 min and 10,000× *g* for 30 min and filtered through a disposable Minisart High Flow filter with a polyestersulfone membrane with a pore diameter of 0.1 microns (Sartorius, Göttingen, Germany, Cat No. 16553K) to remove dead cells and their fragments and were examined by the DLS method for the presence of EPs.

*Methods of statistical processing of measurement results.* Statistical analysis was carried out in R: language and Environment for Statistical Computing (v. 4.0.4; R Core Team, Austria) using the following packages: rstatix (v. 0.7.0.; Alboukadel Kassambara, 2021), ggpubr (v. 0.4.0.; Alboukadel Kassambara, 2020), tidyverse (Wickham et al., 2019), PerformanceAnalytics (Brian G. Peterson., 2020). Differences in the mean values for neighboring groups were analyzed using the Student’s criterion. Pearson correlation coefficient and Pearson partial correlation coefficient [28] were used to assess the statistical significance of the relationship between two continuous variables. The differences were considered statistically significant at *p* ≤ 0.05.

## 3. Results and Discussion

### 3.1. Characteristics of Extracellular Particles

To study EPs produced by human cells in vitro, the culture medium was studied using the DLS method before and after growing Gl-Tr cell line cells in it. It can be seen in Supplement Figure S2A, that in a conditioned medium with fetal bovine serum, three types of particles accumulate after 4 days of cell culture: particles with hydrodynamic diameters (D_h_) 8.0 ± 0.11 nm, 26.4 ± 0.13 nm, and 91.8 ± 0.84 nm.

When cells are cultured in a serum-free medium (Supplement Figure S2B), two types of particles with D_h_ of the order of 25 nm (26.3 ± 0.35 nm) and 90 nm (93.9 ± 0.21 nm) accumulate. At the same time, only one type of particle is present in the pure culture medium with serum (Supplement Figure S2C)—particles with a D_h_ of the order of 8 nm (7.9 ± 0.02 nm). Therefore, the presence of this type of particle is due to its presence in serum samples. Since the main serum protein is albumin, it is safe to say that it is albumin that causes the presence of this peak in Supplement Figure S2A. To prove this assumption, we present the PSD of a chemically pure bovine serum albumin sample (Supplement Figure S2D). Only particles with D_h_ of the order of 8 nm (8.0 ± 0.11 nm) are also present in this figure. This allows us to identify the mentioned type of particle with albumin.

**Figure 1:**
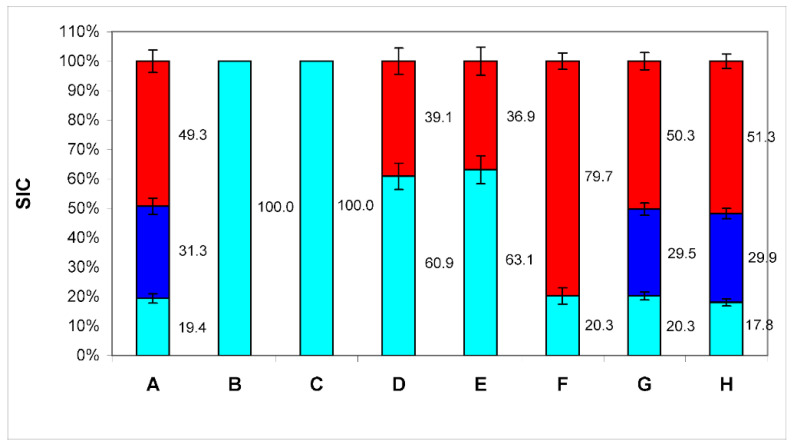
The ratio of the contributions to scattering (SIC) of three particle fractions (BSA—cyan color, exomeres—blue color, and exosomes—red color) in conditioned medium samples after treatment with antibodies bound to protein A immobilized on microspheres. A—SIC of conditioned medium samples; B—SIC of conditioned medium samples after treatment with antibodies bound to protein A immobilized on microspheres to markers CD9; C—SIC of conditioned medium samples, after treatment with antibodies bound to protein A immobilized on microspheres to markers CD63; D—SIC of conditioned medium samples, after treatment with antibodies bound to protein A immobilized on microspheres to markers CD81 in the final concentration of 1 μL of antibodies per 1 mL of medium; E—SIC of conditioned medium samples, after treatment with antibodies bound to protein A immobilized on microspheres to markers CD81 in the final concentration of 2 μL of antibodies per 1 mL of medium; F—SIC of conditioned medium samples, after treatment with antibodies bound to protein A immobilized on microspheres to markers HSP90; G—Negative control 1: SIC of conditioned medium samples, after treatment with antibodies to the CD3 marker; H—Negative control 2: SIC of conditioned medium samples, after treatment protein A without antibodies. The numbers on the graph—SIC of this fraction in %.

Monoclonal antibodies to tetraspanins CD9, CD63, CD81 and heat shock protein HSP90 were added to the investigated medium (at a final concentration of 1 µL of antibodies per 1 mL of medium). After that, protein A from Staphylococcus aureus, immobilized on sepharose microspheres (PrA/S) through covalent bonding, was used for immunoaffinity removal of added antibodies and antigens bound to them (Figure 1B–E). When comparing the data before (Figure 1A) and after treatment with antibodies and protein A (Figure 1B–E), it can be seen that antibodies to CD9 and CD63 markers bind to both types of EPs, and antibodies bind to the HSP90 protein (Figure 1F) only with particles with a D_h_ of the order of 25 nm.

(PrA/S) to markers CD9 (B), CD63 (C), CD81 in the final concentration of 1 μL of antibodies per 1 mL of medium (D) and 2 μL of antibodies per 1 mL of medium (E), HSP90 (F). Negative control: treatment with antibodies to the CD3 marker (G), and microspheres with PrA/S without antibodies (H). The numbers on the graph—SIC of this fraction are in %. Although antibodies to tetraspanin CD81 also bind to both types of EPs, as can be seen from the data (Figure 1D), these antibodies do not remove all particles of the third type from the medium (with a D_h_ of about 90 nm). An increase in the concentration of antibodies to this biomarker twice does not lead to a decrease in the contribution to the scattering of these particles (Figure 1E). So, we are not dealing with a lack of antibodies for stoichiometric binding of all CD81 molecules, but with the absence of this tetraspanin in some particles of this type. Thus, it can be concluded that particles with a D_h_ of about 90 nm are exosomes since they have the appropriate size and carry exosomal biomarkers (for the CD81 biomarker, at least partially).

The HSP90 protein is a protein which has been shown to be detected exclusively on particles with a D_h_ of the order of 25 nm since only such particles are removed by antibodies to this protein (Figure 1F) [10]. Thus, these particles can be identified as exomeres. Although HSP90 is found in the proteome of exosomes, there it is localized exclusively inside the cavity of the particle, and cannot be detected by this method. It is not clear currently why it appears on the surface of exomeres. This issue requires further investigation.

The addition of microspheres with immobilized protein A PrA/S without antibodies (Figure 1H) as negative control and with Anti-CD3 antibodies (Abcam ab5690, Cambridge, MA, USA) (Figure 1G) does not cause changes in PSD conditioned medium with fetal bovine serum after 4 days of cell culture.

Thus, particles with a diameter of the order of 8 nm can be identified as albumin, particles with a D_h_ of the order of 25 nm as exomeres, and particles with a D_h_ of the order of 90 nm as exosomes. Exomeres can be assigned to EPs, as they carry all the characteristic biomarkers (CD 9, CD63, and CD81).

### 3.2. Extracellular Particles and Apoptosis

**Figure 2:**
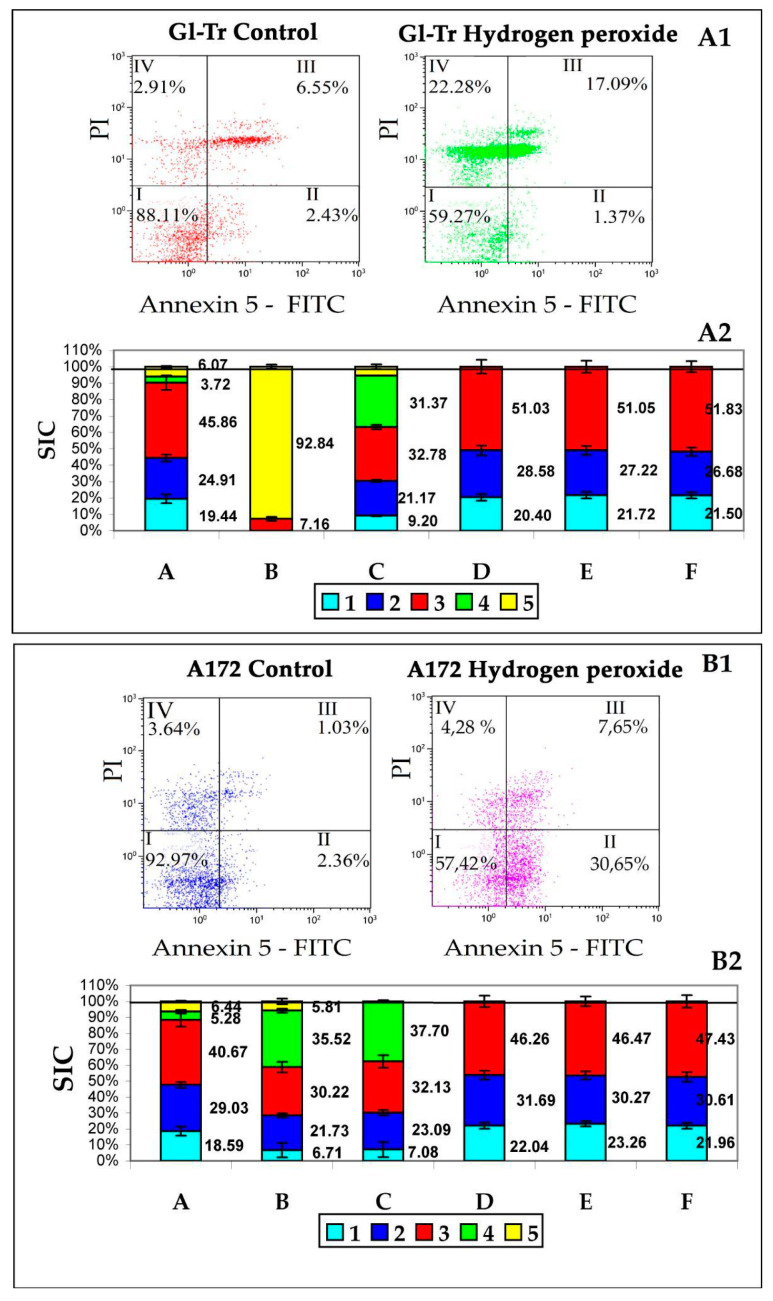
Assessment of the effect of hydrogen peroxide on cells and EPs production.

The effect of hydrogen peroxide on Gl-Tr line cells causes mainly necrotic death. The amount of annexin 5 on the surface of the cell membrane increased in total by only 10%—from 8% to 18.5% (Figure 2A1). In the case of A172 cell line, the picture was somewhat different: we observed the process of classical apoptosis, mainly in the phase of early apoptosis, when there are a lot of cells with an expressed presence of annexin 5 on the membrane, and there are not many cells with membrane damage yet (Figure 2A2).

Panel A1—results of the cytometric test for apoptosis of Gl-Tr line cells. I—living cells (Ann^−^/PI^−^), II—early apoptosis (Ann^+^/PI^−^), III—late apoptosis (Ann^+^/PI^+^), IV—necrosis (Ann^−^/PI^+^). Numbers are the proportion of cells from the total number of analyzed cells (20,000).

Panel B1—results of the cytometric test for apoptosis of A172 line cells. The designations are the same as on panel A1.

Panel A2—the ratio of the contributions to the scattering (SIC) of particle fractions to the total contribution to the scattering of conditioned medium samples (CM) after culturing Gl-Tr line cells. On the *Y*-axis is the scattering intensity contribution (SIC) in %.

Column A is a sample of the original CM without adding H_2_O_2_. Column B is a sample of the original CM after adding H_2_O_2_. Column C is a centrifuged CM sample after adding H_2_O_2_. Column D is a centrifuged CM sample after adding H_2_O_2_ after treatment with PrA/S with antibodies to annexin 5. Column E is a centrifuged sample of the original CM without adding H_2_O_2_ filtered through a filter Minisart High Flow with a membrane with a pore diameter of 0.1 μm. Column F is a centrifuged sample of the original CM after adding H_2_O_2_ filtered through a filter Minisart High Flow with a membrane with a pore diameter of 0.1 μm. Color tags: Blue—albumin, Blue—exomeres, Red—exosomes, Green—apoptotic bodies, Yellow—Fragments of necrotic cells. The numbers on the graph are SIC of the corresponding fraction.

Panel B2—the ratio of the contributions to the scattering (SIC) of particle fractions to the total contribution to the scattering of conditioned medium samples (CM) after culturing A172 line cells. On the *Y*-axis is the scattering intensity contribution (SIC) in %.

The designations are the same as on panel A2.

The results of the study of samples of conditioned medium (CM), after cultivation of cell culture in it, GL-Tr by DLS, as well as the assessment of apoptosis of cells of these cultures by flow cytometry are presented in Figure 2B1. Five types of particles are found in the medium, the first of which can be associated with albumin, the second with exomeres, the third with exosomes. It can be assumed that the green and yellow colors in Figure 2A2,B2 correspond to ApoMV and ApoBD/AB since these particles are removed from the sample by treatment with annexin 5 antibodies immobilized on PrA/S microspheres (column D). It can also be noted that neither exomeres nor exosomes carry annexin 5 on the surface, since they are not removed from the sample by treatment with annexin 5 antibodies immobilized on PrA/S microspheres.

The results of the study of samples of conditioned medium (CM), after cultivation of A172 cell culture in it (Figure 2B2), are basically similar to the results obtained for Gl-Tr cell culture. The differences are mainly due to the different types of death of these lines’ cells. Thus, in columns B and C, the SIC of particles with a size of 550–570 nm, characteristic of classical apoptosis, is pronounced (in green). The main interest in this case is the fact that there are no differences in SIC, both exomeres and exosomes: for Gl-Tr it is 27.2 ± 2.75% against 27.7 ± 2.54% for exomeres (*p*-value = 0.74 (ns)) and 51.1 ± 3.62% against 51.8 ± 3.32% for exosomes (*p*-value = 0.16 (ns)); for A172 it is 30.3 ± 2.5% against 30.6 ± 3.03% for exomeres (*p*-value = 0.31 (ns)) and 46.5 ± 3.06% against 47.4 ± 3.98% for exosomes (*p*-value = 2.99 (ns)).

Thus, for both cell lines, an increase in the yield of both exomeres and exosomes in response to the induction of cell death of both necrotic type and classical apoptosis is not observed.

### 3.3. The Effect of Detergents on the Membranes of Exosomes and Exomeres

The biomechanical properties of membranes depend on the composition and concentration of lipids. Traditionally, the increased rigidity of membranes was associated primarily with the presence of sterols in their composition, mainly cholesterol [23]. The lipid composition of the membranes also determines their resistance to the action of certain ionic detergents. The action of Triton X-100 in its molecular form is aimed at the destruction of the lipid bilayer [29]. The effective concentration of Triton X-100 causing 100% lysis of HeLa cells is 0.2 mmol/L [30]. However, exosomes are somewhat more resistant to the action of this detergent.

**Figure 3:**
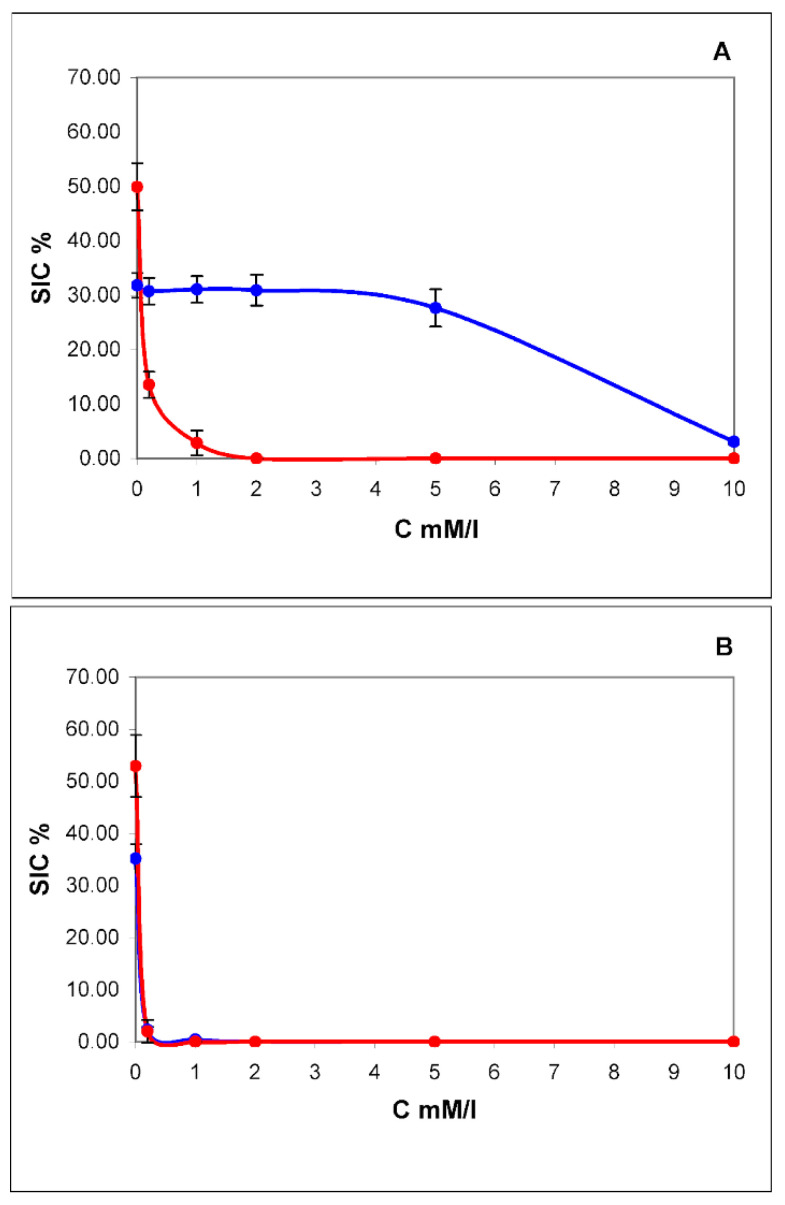
Concentration dependence of lysis of exomeres (blue curve) and exosomes (red curve) by pure Triton X-100 (**A**) and after in a mixture with methylbetacyclodextrin (MβCD) in concentration of 5 mmol (**B**). On the *X*-axis is the concentration of Triton X-100 in mM/L, on the *Y*-axis is the contribution to scattering (SIC) in %.

The dependence of the degree of lysis on the concentration of Triton X-100 for exosomes and exomeres is shown in Figure 3A. From the above data, it can be seen that the sensitivity to Triton X-100 in exosomes is at least an order of magnitude higher than in exomeres. Saponin, on the other hand, acts equally on exomeres and exosomes. Even the minimum concentration 0.08% in both cases gives the maximum effect. This is most likely due to the predominance of sterols, in particular cholesterol, in the lipid spectrum of exomeres. We have attempted to extract cholesterol from exomeres using methylbetacyclodextrin (MβCD). MβCD is a cyclic oligosaccharide that is a selective acceptor of sterols. Beta-cyclodextrins, which include MβCD, have the highest affinity for cholesterol and are the most effective in its extraction from the cell membrane and model membranes [31]. The addition of 5 mmol MβCD to Triton X-100 solution at a minimum concentration acting on exosomes 0.2 mmol/mL leads to almost complete lysis of both exosomes and exomeres (Figure 3B). Thus, it can be argued that cholesterol is an essential part of the lipid composition of exomeres.

### 3.4. Extracellular Particles and Lipoproteins

As shown above (see Section 3.1), there are two types of extracellular particles (EPs) in a conditioned medium: exomeres and exosomes. The average D_h_ of exomeres is 26.4 ± 0.13 nm, and that of exosomes is 91.8 ± 0.84 nm. Both types of these particles contain cholesterol. It has been suggested whether these particles are one or another type of lipoprotein. Exosomes cannot be lipoproteins, since according to [32], lipoproteins have D_h_: HDL—9.7 nm, LDL—19.0 nm, and VLDL—36.3 nm. In the case of exosomes, firstly, they are almost twice as large as the largest lipoproteins—VLDL (D_h_ of exosomes is 91.0 ± 1.78 nm versus 36.8 ± 0.58 nm in VLDL). Secondly, exosomes, unlike lipoproteins, are limited to a bilayer lipid membrane, whereas in lipoproteins the outer layer mainly consists of apolipoprotein proteins. In addition, the floating density of exosomes is 1.13 g/mL [28], whereas the buoyant density of VLDL is 0.96–1.006 g/mL.

So, due to the relative proximity of size and morphology, only LDL could claim the role of exomeres: (19.1 ± 0.18 nm versus 26.3 ± 0.18 nm for exomeres). However, they differ greatly in the buoyant density (1.006–1.063 g/mL for LDL and 1.17–1.19 g/mL for exomeres) [33]. The range of values of buoyant density with exosomes and exomeres corresponds with that of HDL (1.06–1.21 g/mL), but HDL has a much smaller D_h_ (9.7 ± 0.14) versus 26.3 ± 0.18 nm for exomeres or 91.0 ± 1.78 nm for exosomes.

To clarify this aspect, one can check the presence of exosome biomarkers CD 9, CD63, and CD81 and the exomere marker HSP90 on the surface of lipoproteins and ApoA1 and ApoB100 proteins on the surface of exomeres and exosomes. The results obtained are presented in Supplement Tables S1–S3. We have shown that neither VLDL, nor LDL, nor HDL ever carry the CD9, CD63, CD81, and HSP90 markers characteristic of EP, but VLDL and LDL carry the ApoB100 protein characteristic of them, and HDL carries the ApoA1 protein characteristic of it. Exosomes and exomeres, on the contrary, never have ApoA1 and ApoB100 proteins on their surface. Therefore, the exomeres secreted into the culture medium by Gl-Tr culture cells are not lipoproteins.

### 3.5. Dynamics of EPs Production and Cholesterol

**Figure 4:**
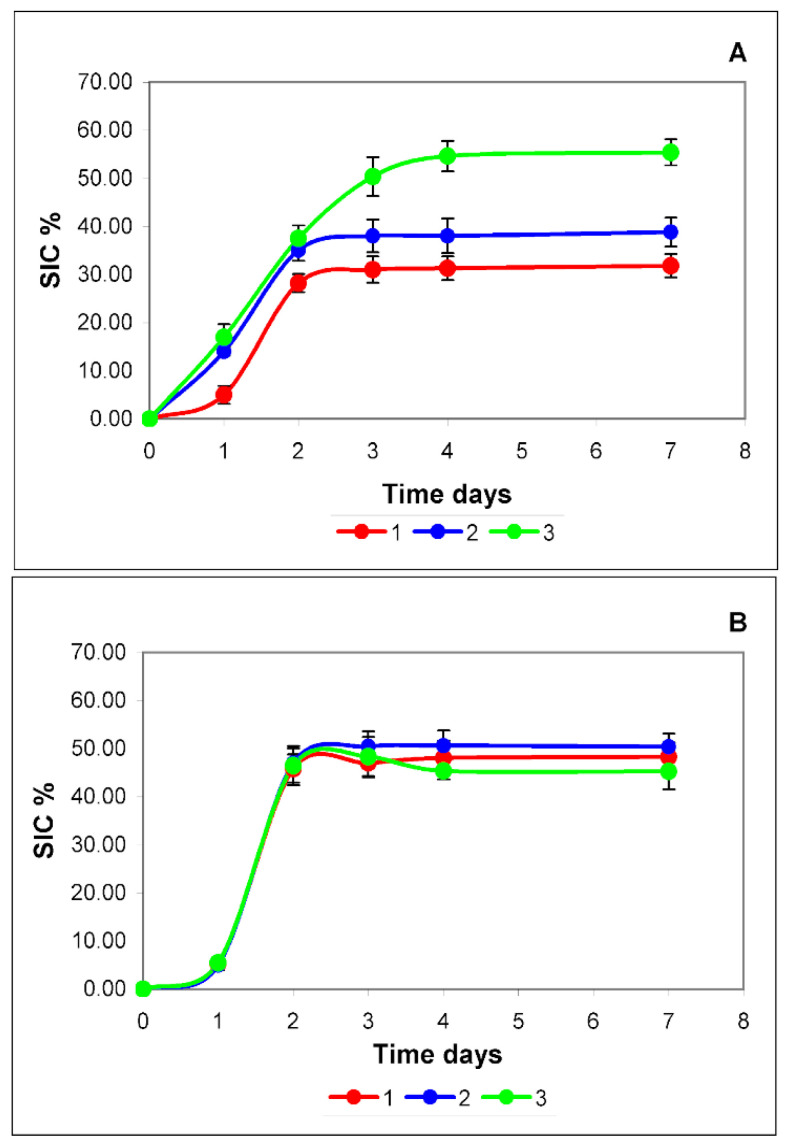
Dynamics of accumulation of exomeres (**A**) and exosomes (**B**) in a conditioned medium sample during cell culture s without the addition of insulin (red curve), with a single (blue curve) and daily (green curve) addition of insulin. On the *X*-axis is the cultivation time in days, on the *Y*-axis is the scattering intensity contribution (SIC) in %. Reliability assessment is in Supplement Figure S4 and Supplement Table S4.

As can be seen from the data given above, cholesterol plays an essential role in the lipid composition and membrane structure of both exosomes and exomeres. It is likely that cholesterol metabolism has a significant effect on the production of EPs by cells. We also tracked the dynamics of exosome and exomere production for 7 days, starting from transplanting to a fresh medium. The results are shown in Figure 4.

Normally (red curves), the accumulation pattern of both types of EPs does not differ qualitatively. During the first two days, there is active production of EPs, then the production of particles stops and the curve reaches a plateau. This type of dynamic is most likely due to the action of negative regulation systems of one of the important components that form both exomeres and exosomes. Cholesterol can be such a limiting factor (red curves in Figure 4A,B).

The daily requirement of cells for cholesterol, in fact, can be covered by biosynthesis. Cholesterol biosynthesis begins with acetyl-CoA. The carbon skeleton of sterol is constructed in a long and complex sequence of reactions. In the first stage, mevalonate is formed from three acetyl-CoA molecules. At this stage, when the enzyme 3-HMG-CoA reductase is activated by phosphorylation (effectors: insulin, thyroxine), cholesterol biosynthesis is regulated. Further stages are of no interest to us. It is essential that the addition of insulin to the culture medium should enhance intracellular cholesterol synthesis. This should lead, if cholesterol plays a significant role in the production of exosomes or exomeres, to an increase in the yield of these EPs [26,27].

In our experiments, we used the dosage form of insulin detemir, the drug Levemir. The duration of the drug is up to 24 h, which provides the possibility of single daily administration. The administration of insulin was carried out in two modes: once—immediately before cell culture; and daily—immediately after sampling the culture medium for research.

The curve of insulin single administration mode (blue curve) for exosomes (Figure 4B) practically coincides with the curve of production dynamics without insulin administration (red curve). The dynamics of exosome accumulation both after a single administration of insulin and during its daily administration (green curve) are almost identical and do not differ from the dynamics of accumulation of these particles in the medium without insulin (Figure 4B). Quantitative differences, in this case, are not statistically significant.

In the case of exomeres (Figure 4A), insulin causes an increase in the cellular production of these particles. With a single injection, this effect is observed during the first two days (blue curve). The differences are statistically significant.

With a daily regime of insulin administration (green curve), the dynamics of exomeres accumulation (Figure 4A) for 4 days significantly differs from both a single regime and the dynamics of exosome accumulation (Figure 4B). For 4 days, a linear increase in the accumulation of exomeres is observed, and only then does the curve reach a plateau. The plateau may be associated with the depletion in the culture medium of the main component for the synthesis of cholesterol—acetyl-CoA or raw materials for its synthesis.

So, it can be stated that cholesterol can play a significant role in the production of exomeres by cells and does not affect the production of exosomes.

### 3.6. EVs and Cholesterol In Vivo

The observed connection of the secretion of exomeres with cholesterol metabolism suggests that they, among other things, can perform the function of excretion of excess cholesterol from cells. To confirm this statement, we investigated the correlations of the number of exosomes and exomeres in blood plasma with the concentration of total cholesterol and cholesterol bound by lipoproteins of very low (VLDL-C), low (LDL-C), and high (HDL-C) density.

We examined the plasma of 41 donors using the DLS method. Donors were selected from patients of the Pavlov St. Petersburg State Medical University polyclinic who received a referral from their attending physician for lipid profile analysis. The group of donors was specially formed to be heterogeneous to obtain the maximum spread of the studied parameters. The statistical processing data are given in Supplement Table S5. When analyzing the data obtained, it turned out that the distributions of values of almost all parameters differ from the normal distribution. Only the hydrodynamic diameters of exomeres and exosomes are close to the normal distribution. All other distributions were strongly asymmetric (the asymmetry is 0.6–08, which is significantly higher than the significance limit of 0.5). This is due to the principle of forming a group of volunteers. As described in the materials and methods, the survey data were divided into three groups: a control group, groups with prescribed correction of cholesterol concentration before correction (“Before therapy”) and after correction (“After therapy”).

**Figure 5:**
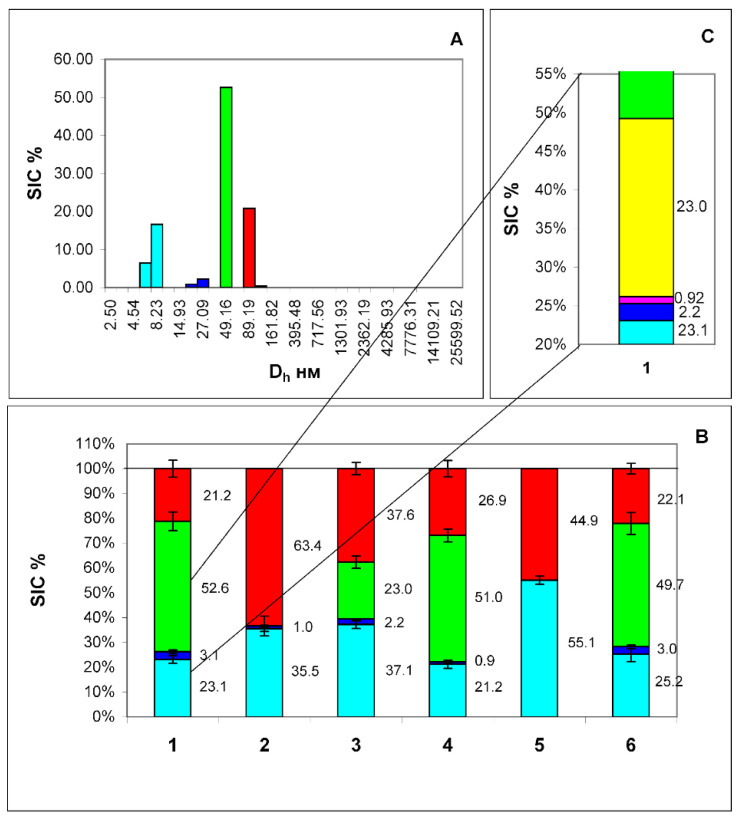
Histogram of particle size distribution in filtered plasma from a healthy volunteer (**A**). The axes are the same as in Figure 1. Histogram of the distribution of contributions to the total scattering of four particle fractions: (human serum albumin (HSA)—cyan color, exomeres—blue color, exosomes—red color, and particles of unknown etiology—green color) (**B**), corresponding to the peaks of the histogram (**A**). 1—filtered sample of the original plasma, 2—the same after adding Protein A immobilized in sepharose microspheres (PrA/S) with antibodies to the CD9 marker, 3—the same after adding PrA/S with antibodies to the HSP90 marker, 4—the same after adding PrA/S with antibodies to the marker ApoB100, 5—the same after the addition of PrA/S with antibodies to the ApoB100 and CD9 markers, 6—the same after the addition of PrA/S with antibodies to the CD3 marker. (**C**)—Detailed display of 2 heterogeneous fractions of sample 1, (blue and green) including exomeres (blue color), LDL (magenta color), VLDL (yellow color), and particles of unknown etiology (green color). The numbers on the graph are the scattering intensity distribution (SIC) of this fraction in %. From this fact, it can be concluded that this fraction is not homogeneous and consists of exomeres and LDL particles. The results of the Student *t*-test indicate that there are significant differences between the contribution to scattering of a fraction of particles with a D_h_ of about 26 nm and the same fraction after treatment with antibodies to the ApoB100 biomarker (exomeres without LDL particles). In all groups, the *p*-value was less than 0.0001.

**Figure 6:**
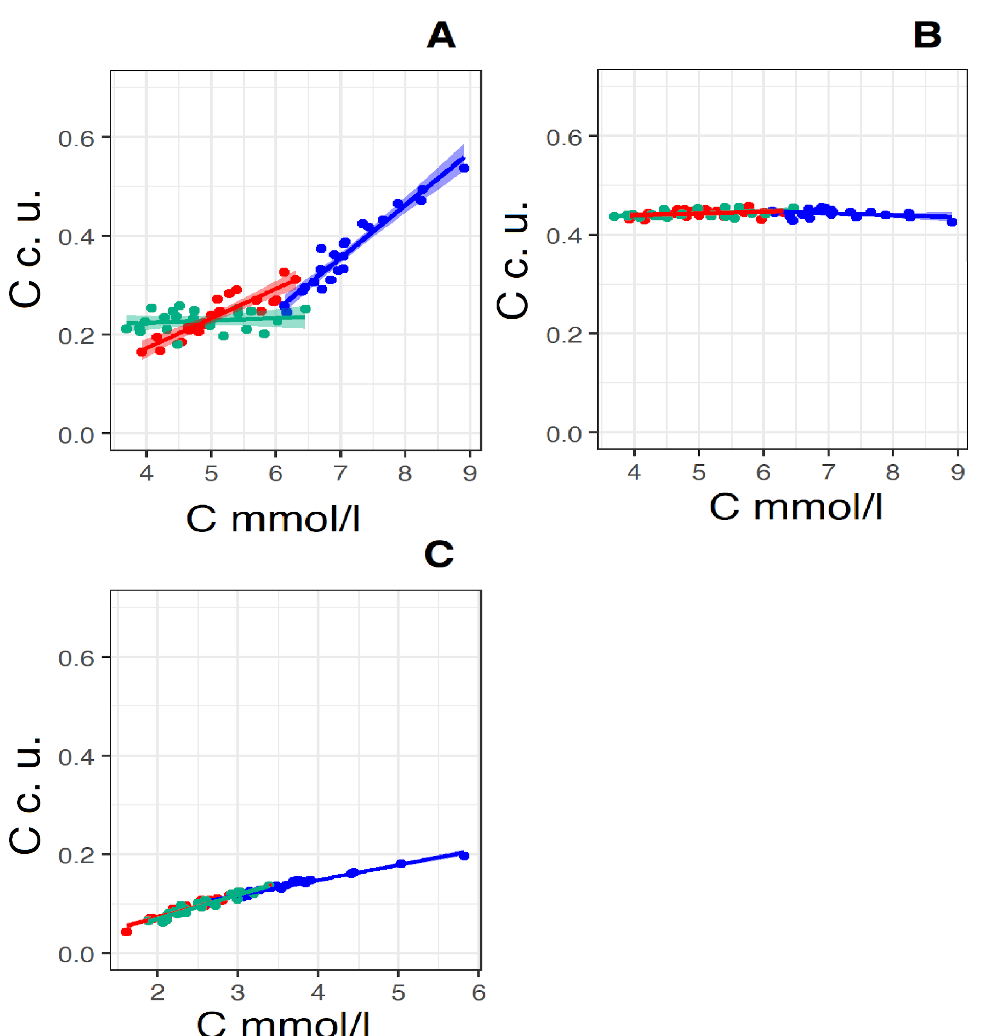
Linear regression lines of the pairs: “total cholesterol concentration (*X*-axis) and relative concentration of exomeres (*Y*-axis)” (**A**), “total cholesterol concentration (*X*-axis) and relative concentration of exosomes (*Y*-axis)” (**B**) and “total cholesterol concentration (*X*-axis) and relative concentration of LDL” (*Y*-axis) (**C**) (*X*-axis in mmol/L, *Y*-axis in convention units—c. u., which is proportional square root of SIC) in the control group (Red straight N = 19) and in the groups “Before therapy” (Blue straight N = 22) and “After therapy” (Green straight N = 22). For pear “total cholesterol concentration and relative concentration of exomeres” in the control group linear regression equitation: y = 0.06x + 0.07, R2 = 0.81, *p* < 0.001, in the group “Before therapy” linear regression equitation: y = 0.11x − 0.39, R2 = 0.92, *p* < 0.001, in the group “After therapy” linear regression equitation: y = 0.005x + 0.20, R2 = 0.02, *p* < 0.01. For pear “total cholesterol concentration and relative concentration of exosomes” in the control group linear regression equitation: y = 0.035x + 0.43, R2 = 0.053, *p* < 0.01, in the group “Before therapy” linear regression equitation: y *=* 0.039x − 0.47, R2 = 0.082, *p* < 0.05, in the group “After therapy” linear regression equitation: y = 0.041x + 0.42, R2 = 0.16, *p*—ns (>0.05). For pear “total cholesterol concentration and relative concentration of LDL” in the control group linear regression equitation: y *=* 0.045x − 0.018, R2 = 0.94, *p ≤* 0.0001, for group “Before therapy” linear regression equitation: y = 0.031x + 0.025, R2 = 0.097, *p ≤* 0.0001, for group “After therapy” linear regression equitation: y *=* 0.048x − 0.027, R2 = 0.90, *p*—ns (>0.001).

In the filtered plasma by the DLS method, we registered four standard sizes of diffusers (Figure 5A). The smallest particles (D_h_ = 7.8 ± 0.14 nm) can be associated with albumin. Particles having D_h_ 26.2 ± 0.63 nm may consist of exomeres (D_h_ 26.4 ± 0.13 nm) and low-density lipoproteins (19.1 ± 0.18 nm). It was previously shown that these particles carry the biomarkers CD9, CD63, CD81 and the marker of exomeres HSP90 on the surface [13]. In addition, particles from this size range (blue in the histogram Figure 5B) are partially removed by microspheres with PrA/S carrying antibodies to CD9 and HSP90 biomarkers (exomeres) (Figure 5B-2,B-3), and partially by antibodies to the low-density lipoprotein biomarker ApoB100 (Figure 5B-4), and are completely removed by a mixture of CD9 and ApoB100 markers (Figure 5B-5). This is shown in more detail in Figure 6C. The blue color on the histogram corresponds to exomeres, and magenta corresponds to LDL particles.

Particles with a D_h_ of 90.2 ± 2.38 nm are identified as exosomes since it has been shown that these particles carry the biomarkers CD9, CD63, CD81 on the surface and do not carry the marker of exomeres HSP90 [10]. They are removed only by antibodies to the CD63 marker and a mixture of antibodies to the CD63 and ApoB100 markers (Figure 5B-2,B-5) (red color on the histogram Figure 5B). Finally, there are particles of unknown nature in the plasma having a D_h_ of 49.2 ± 2.13 nm. They are partially removed only by antibodies to the ApoB100 marker, hence, very-low-density lipoproteins carrying this marker on the surface form part of this particle fraction. This is also reflected in Figure 5C, where the VLDL particles are marked in yellow.

The characteristic of the distribution of the parameters of our interest in the groups can be given by the distribution form analysis in the control group and the groups “Before therapy” and “After therapy”. To do this, the asymmetry and kurtosis of the distribution are calculated. The calculation results are shown in Supplement Tables S5 and S6. Distributions of all parameters except VLDL cholesterol in all three groups are characterized by insignificant asymmetry (<0.25) and kurtosis close to zero.

From the data given in the tables, it can be concluded that the data distributions for all parameters, except LDL-C, are close to the normal distribution. Graphically, descriptive statistics in groups are presented in Supplement Figure S5.

From the data in the figure, it can be seen that for SIC exomeres, total cholesterol, LDL-C, and HDL-C, there are reliable differences between the group “Before therapy” and the other groups, and there are no significant differences between the control group and the group “After therapy”, which indicates the success of the therapeutic effect. At the same time, the presence of reliable differences between the control group and the “After therapy” group for SIC exosomes and VLDL-C cholesterol, and especially the absence of significant differences between the “Before therapy” and “After therapy” groups for VLDL-C, means that taking rosuvastatin at a dose of 10 mg/day does not affect the values of these parameters.

In Table 1, Table 2 and Table 3 and Supplement Tables S7–S9, the matrices of correlation pairs of lipid profile and SIC parameters for exomeres and exosomes in the three above-mentioned groups are presented. A strong correlation will be considered a correlation with R ≥ 0.45, an average correlation if 0.25 ≤ R < 0.45, and a weak correlation if R < 0.25. In the control group (Table 1), a strong positive Pearson correlation was observed for a pair of SIC exomeres—total cholesterol concentration R = 0.98 *p* = 0.0001. At the same time, the partial correlation between these parameters is weaker, although it remains rather strong (R = 0.46, *p* = 0.001). Strong Pearson correlations are also observed in the pairs: SIC exomeres and LDL-C concentration (R = 0.77, *p* = 0.0001) and SIC exomeres and LDL-C (R = 0.47, *p* = 0.05). In the pair of SIC exomeres and HDL-C Pearson, correlation was moderate (R = 0.38, *p* = ns). Still, the partial correlations in all these pairs are weak. Strong Pearson correlations are due to strong partial correlations in pairs: total cholesterol concentration—cholesterol concentration in lipoproteins. (R from 0.82 to 0.47). Strong partial correlations between total cholesterol and all types of lipoproteins are expected and serve as confirmation of the correctness of the method. Negative values of correlation coefficients for exomeres in pairs of total cholesterol and LDL-C, total cholesterol and VLDL-C, and total cholesterol—HDL-C in the “Before therapy” group may indicate a competitive inhibition of the process of lipoprotein formation by synthesis of exomeres and in the cell. (See Table 1).

A similar pattern is typical for the “After therapy” group (Table 3), although on average the values of both the Pearson correlation coefficients and the coefficients of partial correlations are lower. In the “Before therapy” group (Table 2), on the contrary, there are strong partial correlations both between SIC and total cholesterol concentration (R = 0.95, *p* = 0.0001) and between SIC exomeres and cholesterol concentration in lipoproteins. In addition, as in the control group, there are strong correlations between the concentration of total cholesterol and the concentration of cholesterol in lipoproteins.

Thus, a strong partial correlation in all three groups is observed only in the SIC exomeres—total cholesterol pair. In the case of exosomes, the processes of their formation in none of the groups correlate with the cellular processes in which cholesterol is involved. For exosomes, (Supplement Tables S7–S9) partial correlations for all pairs of parameters are either unreliable or weak. Strong or average reliable Pearson correlations can be explained by the fact that the SIC values for exomeres and exosomes are interrelated quantities, and an increase in one of them automatically leads to a decrease in the other, since the total contribution to the scattering of all fractions of scatterers is always 100%. Therefore, in this and other similar cases, the use of partial correlations is more justified.

Figure 6 shows the linear regression lines of a pair of parameters, total cholesterol—particle concentration. It is known that with an equal contribution to scattering (SIC), the number of particles of a given size is proportional to the 6th power of the size (R_h_/D_h_) of the scatterers [34], or the square of the concentration of the scatterers, and therefore the concentration of the scatterers is proportional $\sqrt{SIC}$. That is, on the *X*-axis, the concentration of total cholesterol in mmol/L is deposited on these graphs, and on the *Y*-axis, the concentration of particles in conventional units is directly proportional $\sqrt{SIC}$.

Figure 6A shows regression lines for the total cholesterol—exomere concentration pair. The peculiarity of this pair is the different slope of the regression lines for the control group and the group “Before therapy”; the slope coefficient of the direct group “Before therapy” is almost twice the coefficient of the control group. It can be assumed with high probability that in the case of the control group there are two processes competing for cholesterol: the formation of lipoproteins and the synthesis of exomeres. With an increase in cholesterol concentration, there comes a time when the limiting factor of lipoprotein synthesis is not a lack of cholesterol, but a lack of apolipoproteins. For LDL and VLDL, it is apolipoprotein Apo-B100, and for HDL—Apo-A1. This moment occurs when the concentration of total cholesterol in plasma is about 6–6.5 mmol/L. This assumption is supported by the fact that the regression lines intersect in this concentration range and the slope coefficients of the regression lines for the pair “total cholesterol concentration—LDL particle concentration” coincide with an accuracy of 3 decimal places (Figure 6C). Blocking cholesterol synthesis with rosuvastatin leads to the cessation of exomere production—the slope coefficient of the regression curve is 0.005, and the process of LDL synthesis continues due to cholesterol coming from food—the slope coefficient of the regression curve is 0.05 (Figure 6C).

For the total cholesterol–exosome concentration pair (Figure 6B), the regression lines for all three groups are almost parallel to the *X*-axis. That is, the processes leading to an increase in cholesterol concentration and the process of synthesis of exosomes are independent. This is also evidenced by the low degree of correlation between the concentration of total cholesterol and the concentration of exosomes in plasma (see Table 3, Supplement Tables S7 and S8). A high degree of correlation in the “Before therapy” group is not taken into account, since it is not reliable (*p* > 0.05).

**Figure 7:**
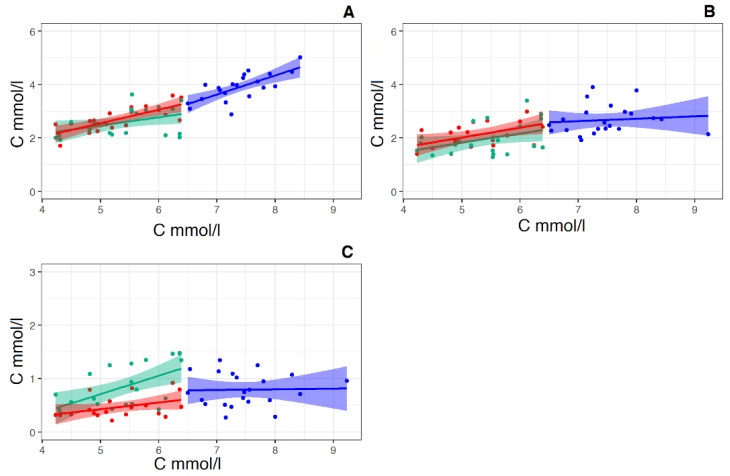
Linear regression of the pairs “total cholesterol concentration (*X*-axis)—calculated LDL-C concentration (*Y*-axis)” (**A**), “total cholesterol concentration (*X*-axis)—HDL-C concentration (*Y*-axis) pairs” (**B**) and the pairs “total cholesterol concentration (*X*-axis)—calculated VLDL-C concentration (*Y*-axis)” (**C**), (*X*-axis and *Y*-axis in mmol/L) in the control group (Red straight, N = 19) and in the groups “Before therapy” (Blue straight N = 22) and “After therapy” (Green straight N = 22). For pear “total cholesterol concentration—calculated LDL-C concentration” for the control group linear regression equitation: y = 0.51x + 0.02, R2 = 0.53, *p* < 0.001. For the group “Before therapy” linear regression equitation: y = 0.71x—1.36, R2 = 0.49, *p* < 0.001. For group “After therapy” linear regression equitation: y = 0.31x + 0.9, R2 = 0.13, *p* < 0.05. For pear “total cholesterol concentration—calculated HDL-C concentration for the control group linear regression equitation: y = 0.37x + 0.18, R2 = 0.22, *p* < 0.01. For the group “Before therapy” linear regression equitation: y = 0.09x + 1.98, R2 = 0.038, *p* < 0.05. For the group “After therapy” linear regression equitation: y = 0.35x + 0.92, R2 = 0.13, *p*—ns (> 0.05). For pear “total cholesterol concentration—calculated VLDL-C concentration for the control group linear regression equitation: y = 0.12x − 0.20, R2 = 0.11, *p* < 0.05. For the group “Before therapy” linear regression equitation: y = 0.012x + 0.68, R2 = 0.049, *p* < 0.05. For the group “After therapy” linear regression equitation: y = 0.34x − 0.99, R2 = 0.11, *p*—ns (> 0.05).

In the total cholesterol—LDL particle concentration pair (Figure 6C), the regression lines for all three groups practically form one common line (the values of the slope coefficients of the regression lines differ only in the third decimal place). An increase in the concentration of LDL particles with an increase in cholesterol concentration increases directly proportionally over the entire range of cholesterol concentrations measured by us, both in the control group and in the “Before therapy” group. Most likely, the concentration of cholesterol per 1 LDL particle is close to a constant, and an increase in the concentration of LDL-C in plasma occurs due to an increase in the number of LDL particles. The fact that the regression line for the “After therapy” group also falls on the general line can be explained by the presence of two methods of cholesterol intake in the body: in addition to the pathway of cholesterol synthesis in cells, which is blocked by rosuvastatin, there is a method of cholesterol intake into the body with food. When taking rosuvastatin, the concentration of LDL particles in plasma decreases, which is reflected in a drop in the concentration of cholesterol in plasma (Figure 7C).

The regression direct pairs “total cholesterol concentration—calculated HDL-C “ and “total cholesterol concentration—VLDL-C” behave exactly the same (Figure 7B,C). In this case, too, the values of the slope coefficients of the curves in the group before therapy are insignificant. That is, the increase in total cholesterol in this group is a consequence of an increase in LDL-C, in which the coefficient of the slope of the regression curve in the group “Before therapy” is 0.71 (Figure 7A). It is also worth noting that rosuvastatin reduces the concentration of both “bad” LDL and “good” HDL cholesterol with the same intensity.

At the same time, the direct linear regression of the concentration of the pair “total cholesterol concentration—calculated LDL-C concentration” (according to Friedewald formula) [35] (Figure 7A) significantly differs from both the previous pair and the pair of total cholesterol concentration and LDL particle concentration (see Figure 6C). These straight lines no longer form a common straight line, and the slope coefficient of the regression line group “Before therapy” is 1.5 times greater than that of the regression line for the control group (Figure 7A). In this case, the course of regression lines resembles that of the pair “total cholesterol concentration and relative exomeres concentration” (Figure 6A).

Recall that Friedewald’s formula [36] implies that the carriers of cholesterol in plasma are exclusively lipoproteins, and does not take into account cholesterol contained in other extracellular particles, in particular exomeres.

We have shown that exomeres not only contain cholesterol in their composition but are also produced by cells for the excretion of excess intracellular cholesterol. Since the result of the calculation according to the Friedewald formula is the concentration of LDL-C, all non-lipoprotein cholesterol will be counted as LDL-C. This explains the similarity of regression lines in pairs: total cholesterol—the relative concentration of exomeres (Figure 6A) and “Total cholesterol—calculated LDL-C “ (Figure 7A). In addition, the relatively steep slope of the regression lines in the control group for both the pair “total cholesterol concentration and relative exomere concentration” (Figure 6A), and for the pair “Total cholesterol—calculated LDL-C” (Figure 7A) may indicate in favor of the assumption that the removal of cholesterol from the cell is a two-stage process, in the first stage of which cholesterol is removed from the cell in the form of LDL particles, and in the second—in the form of exomeres.

## 4. Conclusions

1. Cholesterol is an essential part of the lipid composition of exomeres.
2. Cholesterol can play a significant role in the production of exomeres by cells and does not affect the production of exosomes.
3. Exomeres secreted into the culture medium by GL-Tr culture cells are not lipoproteins.
4. It can be suggested that exomeres are produced by cells to excrete excess intracellular cholesterol. The process of producing exomeres in this case is regulated by the concentration of intracellular cholesterol, which in turn depends on the rate of the process of producing lipoproteins (in particular LDL). The process of producing exomeres, apparently, is inducible and starts when a certain threshold concentration of intracellular cholesterol is reached.
5. In vivo exomeres, unlike exosomes, can play a significant role in the processes of cholesterol excretion from cells.

Thus, it can be concluded that exomeres play an essential role in the transport of cholesterol in blood plasma. Moreover, these effects are observed not only in vitro but in vivo as well.

## Figures and Tables

**Table 1:** Pearson correlation and partial correlation coefficients of lipid profile parameters and SIC exomeres and in the control group.

	SIC Exomeres	Cholesterol	LDL-C	VLDL-C	HDL-C
Exomeres		0.93 ″ (****)	0.77 ″ (****)	0.47 ″ (*)	0.38 ′ (ns)
Cholesterol	0.46 ″ (****)		0.80 ″ (****)	0.49 ″ (*)	0.41 ′ (ns)
LDL-C	0.21 (ns)	0.72 ″ (*****)		0.59 ″ (*)	−0.14 (ns)
VLDL-C	−0.02 (ns)	0.50 ″ (**)	−0.34 ′ (ns)		−0.28 ′ (ns)
HDL-C	0.15 (ns)	0.74 ″ (*****)	−0.84 ″ (*****)	−0.60 ″ (***)	

ns: *p* > 0.05, *: *p* ≤ 0.05, **: *p* ≤ 0.01, ***: *p* ≤ 0.001, ****: *p* ≤ 0.0001, *****: *p* << 0.0001. Cyan color—Pearson correlation, light green color—partial correlation. ″—strong correlation, ′—medium correlation, no marks—weak correlation.

**Table 2:** Pearson correlation and partial correlation coefficients of lipid profile parameters and SIC exomeres group “Before therapy”.

	SIC Exomeres	Cholesterol	LDL-C	VLDL-C	HDL-C
Exomeres		0.98 ″ (****)	0.75 ″ (****)	0.19 (ns)	0.05 (**)
Cholesterol	0.95 ″ (*****)		0.75 ″ (***)	0.19 (ns)	0.08 (****)
LDL-C	0.82 ″ (*****)	−0.81 ″ (****)		0.34 ′ (ns)	−0.50 ″ (*)
VLDL-C	0.47 ″ (ns)	−0.45 ″ (**)	−0.085 (ns)		−0.60 ″ (ns)
HDL-C	0.73 ″ (**)	−0.86 ″ (*****)	−0.53 ″ (*)	−0.22 (*)	

ns: *p* > 0.05, *: *p* ≤ 0.05, **: *p* ≤ 0.01, ***: *p* ≤ 0.001, ****: *p* ≤ 0.0001, *****: *p* << 0.0001. Cyan color—Pearson correlation, light green color—partial correlation. ″—strong correlation, ′—medium correlation, no marks—weak correlation.

**Table 3:** Pearson correlation and partial correlation coefficients of lipid profile parameters and SIC exomeres in the group “After therapy”.

	SIC Exomeres	Cholesterol	LDL-C	VLDL-C	HDL-C
Exomeres		0.545 ″ (***)	0.28 ′ (ns)	0.19 (ns)	0.10 (ns)
Cholesterol	0.51 ″ (*****)		0.36 ′ (ns)	0.40 ′ (ns)	0.16 (ns)
LDL-C	0.01 (****)	0.40 ′ (****)		0.40 ′ (ns)	−0.38 ′ (ns)
VLDL-C	−0.08 (ns)	0.38 ′ (ns)	0.08 (ns)		−0.33 ′ (ns)
HDL-C	0.03 ′ (***)	0.37 ′ (****)	−0.41 ′ (*)	−0.35 ′ (ns)	

ns: *p* > 0.05, *: *p* ≤ 0.05, ***: *p* ≤ 0.001, ****: *p* ≤ 0.0001, *****: *p* << 0.0001. Cyan color—Pearson correlation, light green color—partial correlation. ″—strong correlation, ′—medium correlation, no marks—weak correlation.

## Data Availability

The data presented in this study are available on request from the corresponding author.

## Supplementary Materials

The following supporting information can be downloaded at: https://www.mdpi.com/article/10.3390/membranes12060618/s1, **Figure S1.** Dynamics of changes in contributions to scattering (SIC—scattering intensity distribution) of 3 particle fractions (BSA—cyan colour, exomeres—blue colour and exosomes—red colour) in conditioned medium samples depending on the cultivation time of a monolayer of cells after changing the culture medium; **Figure S2.** Histograms of particle size distribution (PSD) in a conditioned medium with the addition of serum (A) and without the addition of serum (B) after 4 days of cultivation of Gl-Tr cell culture, pure culture medium (C) and bovine serum albumin solution (D); **Figure S3.** Particles size distribution (PSD) of conditioned media samples. On the X-axis is the hydrodynamic diameter of the particles (D_h_) in nm, on the Y-axis is the contribution of particles of a given size to the total scattering of the sample (SIC) in %; **Table S1.** Identification of biomarkers by means of DLS combined with immunoprecipitation on HDL standard sample particles; **Table S2.** Identification of biomarkers by means of DLS combined with immunoprecipitation on LDL standard sample particles; **Table S3.** Descriptive statistics of the results of exomeres and exosomes from plasma samples measurement by DLS method and lipid profile data from volunteers who participated in the studies (N=41); **Figure S4.** The reliability of the data shown in Figure S5. A—exomeres, B—exosomes, red color—control, blue color—single addition of insulin to the culture medium, green color—daily addition of insulin to the culture medium. At the top is the *p*-value score. ns: *p* > 0.05; *: *p* ≤ 0.05; **: *p* ≤ 0.01; ***: *p* ≤ 0.001; ****: *p* ≤ 0.0001; **Table S4.** *p*-value values in pairs of graphs in Figure S5; **Table S5.** Asymmetry of distribution of measurements by the DLS method results for exomeres and exosomes from plasma samples and lipid profile data in three groups; **Figure S5.** Descriptive statistics of contributions to the dispersion of exomeres, exosomes and lipid profile data in three groups of volunteers who participated in the study; **Table S6.** The kurtosis of the distribution of the results of measurement by the DLS method of exomeres and exosomes from plasma samples and lipid profile data in three groups; **Table S7.** Pearson correlation and partial correlation coefficients of lipid profile parameters and SIC exosomes in the control group; **Table S8.** Pearson correlation and partial correlation coefficients of lipid profile parameters and SIC exosomes in the group “Before therapy”; **Table S9.** Pearson correlation and partial correlation coefficients of lipid profile parameters and SIC exosomes in the group “After therapy”.
