# Supplementary material for: Extracellular Particles as Carriers of Cholesterol Not Associated with Lipoproteins
Source: Membranes (Basel). 2022 Jun 14;12(6):618. doi: 10.3390/membranes12060618 (PMC9227390; doi:10.3390/membranes12060618)
Supplement: Supplementary file 1 [file membranes-12-00618-s001.zip › membranes-1695617-supplementary.pdf]

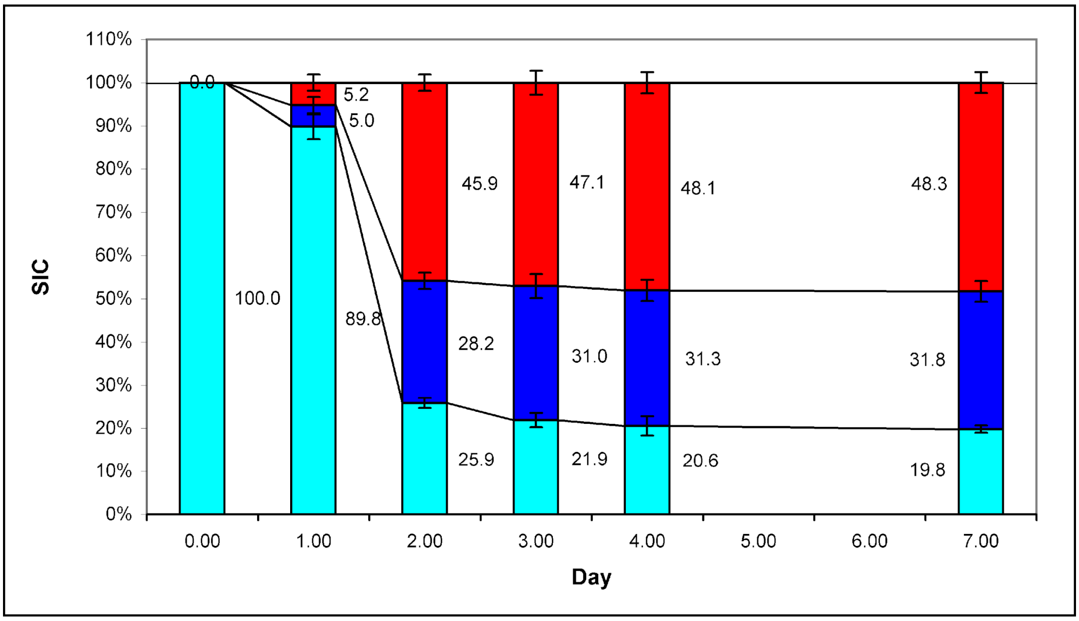

Supplement Figure S1. Dynamics of changes in contributions to scattering (SIC – scattering intensity distribution) of 3 particle fractions (BSA – cyan colour, exomeres – blue colour and exosomes – red colour) in conditioned medium samples depending on the cultivation time of a monolayer of cells after changing the culture medium. On the X-axis is the cultivation time and on the Y-axis – is the contribution to the scattering of the fraction (SIC) to the total scattering of the sample under study. The numbers on the graph – SIC of this fraction in %. It is important to note two things. First, the sum of contributions to the scattering of all fractions of particles is always equal to 100%. The second is that the contribution of exomeres and exosomes to scattering increases, while the contribution of albumin decreases during the first three days after changing the medium, and then both of them reach a plateau. Therefore, in all experiments, except for those where the dynamics of EPs accumulation was studied, CM samples were taken on the 4th day of cultivation.

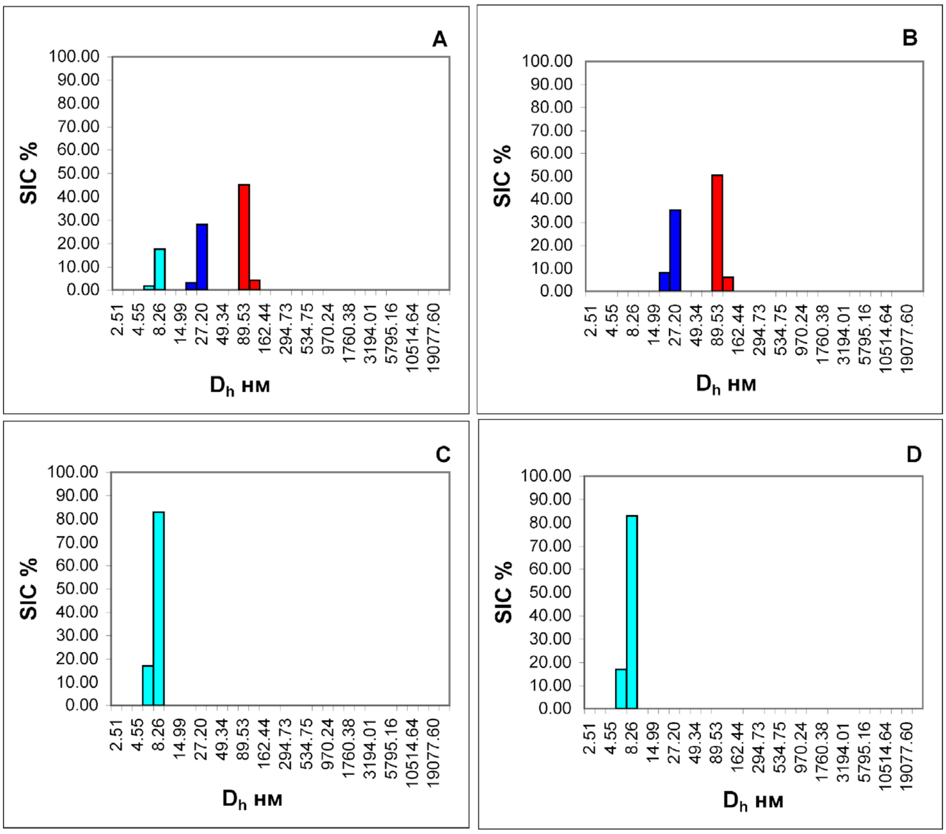

Supplement Figure S2. Histograms of particle size distribution (PSD) in a conditioned medium with the addition of serum (A) and without the addition of serum (B) after 4 days of cultivation of Gl-Tr cell culture, pure culture medium (C) and bovine serum albumin solution (D). On the X-axis is the hydrodynamic diameter of the particles (D_h) in nm, on the Y-axis is the contribution of particles of a given size to the total scattering of the sample (SIC) in %.

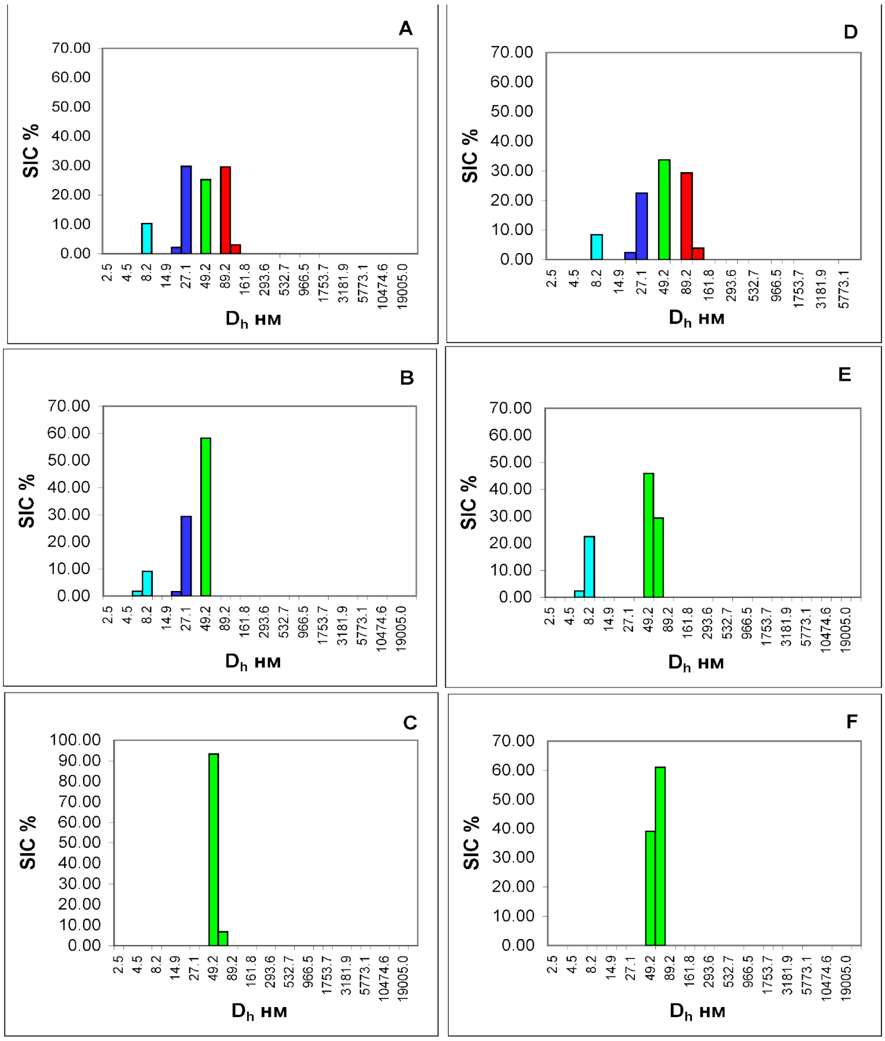

Supplement Figure S3. Particles size distribution (PSD) of conditioned media samples. On the X-axis is the hydrodynamic diameter of the particles (D_h) in nm, on the Y-axis is the contribution of particles of a given size to the total scattering of the sample (SIC) in %.

Panel A - PSD Conditioned medium immediately after addition of Triton X-100 at a final concentration of 2 mM/L

Panel B - PSD of Conditioned medium one hour after incubation with Triton X-100 at 37°C at the same concentration

Panel C - Triton X-100 micelles in PBS are given for control

Panel D - PSD Conditioned medium immediately after adding saponin at a final concentration of 0.08%

Panel E - PSD of Conditioned medium one hour after incubation with saponin at 37°C at the same concentration

Panel F - Saponin micelles in PBS are given for control.

Supplement Table S1. Identification of biomarkers by means of DLS combined with immunoprecipitation on HDL standard sample particles

№	ID	Peak 1		Peak 2		Peak 3		Peak 4	
		D_h nm	SIC %	D_h nm	SIC %	D_h nm	SIC %	D_h nm	SIC %
1	[2]HDL ([1]N=5)	9.5±0.31	100.0	–	–	–	–	–	–
2	[3]HDL + PrA (N=5)	9.8±0.18	99.6±0.28	–	–	–	–	717.6±5*E-14	0.4±0.28
3	[4]HDL + PrA+ AntiCD9 (N=5)	9.5±0.02	99.6±0.37	–	–	–	–	717.6±5*E-14	0.4±0.37
4	[5]HDL +PrA+ AntiCD63 (N=5)	9.7±0.18	99.6±0.30	–	–	–	–	717.6±5*E-14	0.4±0.30
5	[6]HDL + PrA+ AntiCD81 (N=5)	9.7±0.06	99.6±0.36	–	–	–	–	717.6±5*E-14	0.5±0.36

6	[7]HDL + PrA+ AntiHSP90 (N=5)	9.8±0.11	99.7±0.22	–	–	–	–	717.6±5*E-14	0.3±0.22
7	[8]HDL + PrA+ AntiApoA1 (N=5)	–	–	–	–	–	–	787.9±7.48	100
8	[9]HDL + PrA+ AntiApoB100 (N=5)	9.9±0.08	99.7±0.21	–	–	–	–	717.6±5*E-14	0.3±0.21

[1]N − Number of repeated measurements
[2]HDL − HDL standard sample solution
[3]HDL + PrA − HDL standard sample solution and PrA/S (negative control 1)
[4]HDL + PrA+ AntiCD9 − HDL standard sample solution and PrA/S and antyCD9 antibody (see material and methods)
[5]HDL + PrA+ AntiCD63 − HDL standard sample solution and PrA/S and antyCD63 antibody
[6]HDL + PrA+ AntiCD81 − HDL standard sample solution and PrA/S and antyCD81 antibody
[7]HDL + PrA+ AntiHSP90 − HDL standard sample solution and PrA/S and antyHSP90 antibody
[8]HDL + PrA+ AntiApoA1 − HDL standard sample solution and PrA/S and antyApoA1 antibody (positive control)
[9]HDL + PrA+ AntiApoB100 − HDL standard sample solution and PrA/S and antyApoB100 antibody (negative control 2)

Table S2. Identification of biomarkers by means of DLS combined with immunoprecipitation on LDL standard sample particles

№	ID	Peak 1		Peak 2		Peak 3		Peak 4	
		D_h nm	SIC %	D_h nm	SIC %	D_h nm	SIC %	D_h nm	SIC %
1	[2]LDL ([1]N=5)	–	–	19.2±0.43	100.0			–	–
2	[3]LDL + PrA (N=5)	–	–	19.1±0.23	99.6±0.16			717.6±5×10^{-14}	0.4±0.16
3	[4]LDL + PrA+ AntiCD9 (N=5)	–	–	19.1±0. 24	99.6±0.29			717.6±5×10^{-14}	0.4±0.29
4	[5]LDL +PrA+ AntiCD63 (N=5)	–	–	19.0±0.24	99.7±0.24			717.6±5×10^{-14}	0.3±0.24
5	[6]LDL + PrA+ AntiCD81 (N=5)	–	–	19.0±0.16	99.7±0.17			717.6±5×10^{-14}	0.3±0.17
6	[7]LDL + PrA+ AntiHSP90 (N=5)	–	–	19.0±0.18	99.8±0.30			717.6±5×10^{-14}	0.2±0.30
7	[8]LDL + PrA+ AntiApoA1 (N=5)	–	–	19.1±0. 15	99.7±0.17			717.6±5×10^{-14}	0.3±0.17
8	[9]LDL + PrA+ AntiApoB100 (N=5)	–––	–	–	–			764.7±6.96	100

Footnotes are the same as in Table S1

Supplement Table S3. Descriptive statistics of the results of exomeres and exosomes from plasma samples measurement by DLS method and lipid profile data from volunteers who participated in the studies (N=41)

Group	Mean	SD	C_V %	Min.	Q1	Median	Q3	Max.
Exomeres D_h, nm	25.85	0.64	2.48	24.29	25.41	26.00	26.3	26.89
Exomeres SIC %	13.85	7.1	51.26	2.85	7.79	12.74	18.19	29.57
Exosomes D_h, nm	91.07	2.25	2.47	89.66	89.75	90.07	90.42	96.91
Exosomes SIC %	20.15	1.87	9.28	15.88	19.06	20.06	21.8	23.94
LDL SIC %	1.49	0.77	51.43	0.18	0.92	1.37	1.88	3.87
Total cholesterol mmol/l	6.48	1.26	19.44	4.23	5.44	6.54	7.45	9.23
LDL cholesterol mmol/l	3.44	0.89	25.87	1.71	2.66	3.51	3.98	6.14
VLDL cholesterol mmol/l	0.64	0.3	46.88	0.21	0.35	0.57	0.82	1.34
HDL cholesterol mmol/l	2.4	0.57	23.75	1.4	2.03	2.29	2.69	3.9

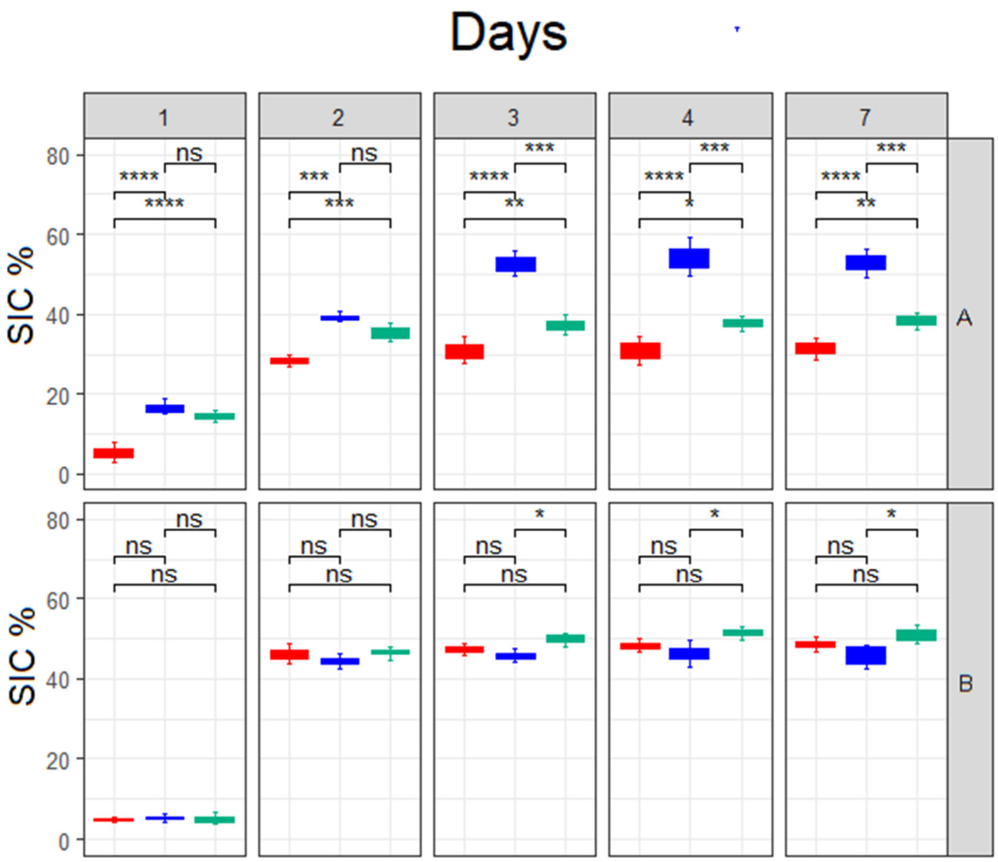

Supplement Figure S4. The reliability of the data shown in Fig.5. A – exomeres, B – exosomes, red color – control, blue color – single addition of insulin to the culture medium, green color – daily addition of insulin to the culture medium. At the top is the p-value score. ns – p > 0.05; – *: p ≤ 0.05; – **: p ≤ 0.01; – ***: p ≤ 0.001; **** – p ≤ 0.0001

Supplement Table S4. P-value values in pairs of graphs in Figure 5

Exomeres (A)					
1 – 2	0.000075 *p < 0.000*[1]	0.00072 *p < 0.001*	0 .00008 *p < 0.0001*	0 .000033 *p < 0.000*[1]	0 .000001 *p < 0.01*
1 – 3	0.000077 *p < 0.000*[1]	0.00088 *p <0.001*	0 .00077 *p < 0.001*	0 .011 *p < 0.05*	0 .00039 *p < 0.01*
2 – 3	0.072 *ns*[1]	0.19 *ns*[1]	0.00035 *p < 0.001*	*0.0001 p < 0.01*	0 .00014 *p < 0.001*
Exosomes (B)					
1 – 2	0 .74 *ns*[1]	0 .55 *ns*[1]	0 .68 *ns*[1]	0 .19 *ns*[1]	0 .19 *ns*[1]
1 – 3	0 .66 *ns*[1]	0 .59 *ns*[1]	0 .12 *ns*[1]	0 .27 *ns*[1]	0 .28 *ns*[1]
2 – 3	0.20 *ns*[1]	0 .25 *ns*[1]	0.044 *p < 0.05*	0.017 *p < 0.05*	0.039 *p < 0.05*

Footnote:

[1] *ns* – not significant

Supplement Table S5. Asymmetry of distribution of measurements by the DLS method results for exomeres and exosomes from plasma samples and lipid profile data in three groups.

Group	Control (N=19)	Before therapy (N=22)	After therapy (N=22)
Exomeres D_h, nm	−0.02	−0.03	0.01
Exomeres SIC %	−0.107	0.01	−0.09
Exosomes D_h, nm	0.02	0.02	−0.01
Exosomes SIC %	0.01	0.00	0.00
LDL SIC %	0.06	0.12	−0.02
Total cholesterol mmol/l	0.00	0.03	−0.04
LDL cholesterol mmol/l	0.13	0.15	0.23
VLDL cholesterol mmol/l	2.36	2.69	0.25
HDL cholesterol mmol/l	−0.05	0.17	−0.08

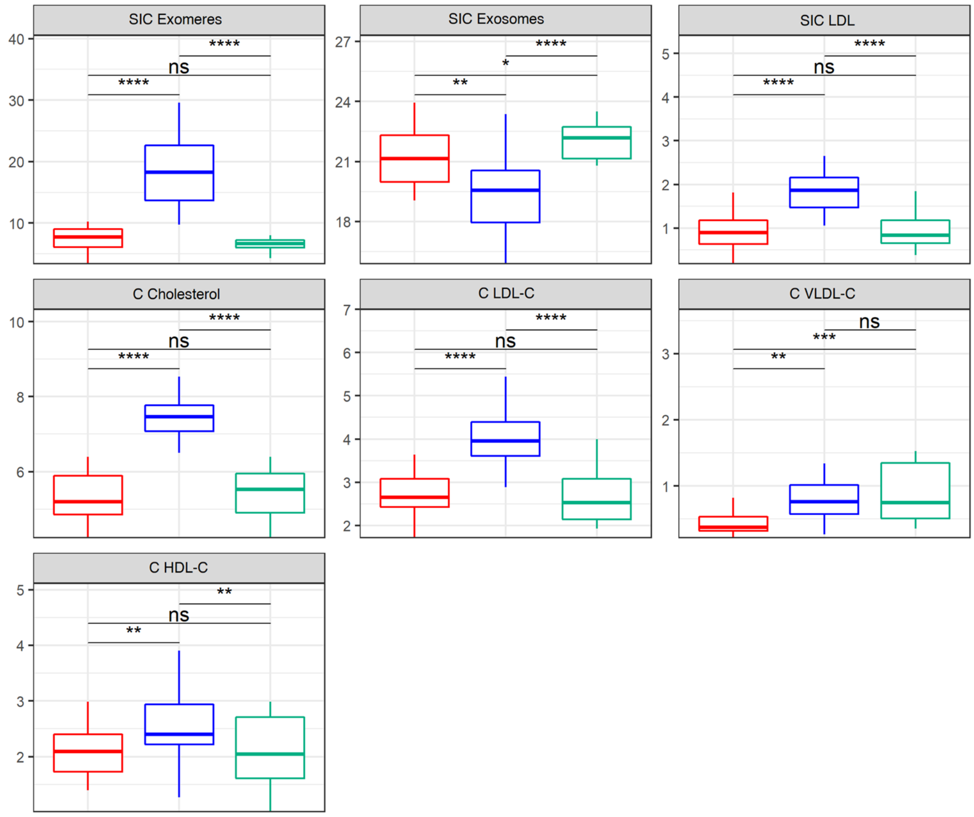

Supplement Figure S5. Descriptive statistics of contributions to the dispersion of exomeres, exosomes and lipid profile data in three groups of volunteers who participated in the study. Control group – red color N =19, Group «Before Therapy» – blue color N = 22, and Group «After Therapy» – green color N = 22. p-value: ns – $p > 0.05$; – *: $p \leq 0.05$; – **: $p \leq 0.01$; – ***: $p \leq 0.001$; **** – $p \leq 0.0001$)

Supplement Table S6. The kurtosis of the distribution of the results of measurement by the DLS method of exomeres and exosomes from plasma samples and lipid profile data in three groups.

Group	Control (N=19)	Before therapy (N=22)	After therapy (N=22)
Exomeres D_h, nm	−0.030	0.026	−0.009
Exomeres SIC %	−0.04	−0.063	0.004
Exosomes D_h, nm	0.018	0.022	0.001
Exosomes SIC %	−0.042	−0.026	−0.068
LDL SIC %	0.056	0.012	−0.018
Total cholesterol mmol/l	−0.022	−0.069	−0.018
LDL cholesterol mmol/l	−0.075	0.018	−0.228
VLDL cholesterol mmol/l	2.110	9.068	−1.974
HDL cholesterol mmol/l	−0.034	0.014	−0.059

Supplement Table 7. Pearson correlation and partial correlation coefficients of lipid profile parameters and SIC exosomes in the control group

	SIC Exosomes	Cholesterol	LDL-C	VLDL-C	HDL-C
SIC Exosomes		-0.40′ (ns)	-0.36′ (ns)	-0.46′′ (ns)	-0.13 (ns)
Cholesterol	0.29′ (ns)		0.80′′ (****)	0.49′′ (*)	0.41′ (ns)
LDL-C	-0.08 (ns)	0.72′′ (*****)		0.59′′(*)	-0.14 (ns)
VLDL-C	-0.39′ (ns)	0.50′′ (**)	-0.34′ (ns)		-0.28′ (ns)
HDL-C	-0.18 (ns)	0.74′′ (******)	-0.84′′ (******)	-0.60′′ (***)	

[1]ns: p > 0.05; – * p <= 0.05; – **: p <= 0.01, – ***: p <= 0.001, – ****: p <= – 0.0001
[2]Cyan color – Pearson correlation, Light green color – partial correlation
[3]′′ – strong correlation, ′ – medium correlation, no marks – weak correlation

Supplement Table 8. Pearson correlation and partial correlation coefficients of lipid profile parameters and SIC exosomes in the group "Before therapy"

	SIC Exomeres	Cholesterol	LDL-C	VLDL-C	HDL-C
SIC Exosomes		-0.57′′ (****)	-0.28′ (****)	0.42′ (ns)	-0.47′′ (**)
Cholesterol	0.20 (ns)		0.74′′ (***)	0.19 (ns)	0.08 (****)
LDL-C	0.10 (ns)	-0.81′′ (***)		0.34′ (ns)	-0.50′′ (*)
VLDL-C	-0.39′ (*)	0.50′′ (ns)	-0.34′ (ns)		-0.60′′ (ns)
HDL-C	-0.-7 (ns)	-0.86′′ (****)	-0.53′′ (*)	-0.22 (ns)	

Footnotes are the same as in Supplement table 7

Supplement Table 9. Pearson correlation and partial correlation coefficients of lipid profile parameters and SIC exosomes in the group "After therapy"

	SIC Exomeres	Cholesterol	LDL-C	VLDL-C	HDL-C
SIC Exosomes		0.23 (ns)	-0.30′ (ns)	-0.041 (ns)	0.17 (ns)
Cholesterol	0.46′′ (ns)		0.36′ (ns)	0.40 (ns)	0.16 (ns)
LDL-C	-0.38′ (ns)	0.40′ (ns)		0.40′ (ns)	-0.38′ (ns)
VLDL-C	-0.08 (ns)	0.38′ (ns)	0.08 (ns)		-0.33′ (ns)
HDL-C	-0.09 (ns)	0.37′ (****)	-0.41′ (*)	-0.35′ (ns)	

Footnotes are the same as in Supplement table 7
